# Continuous Epidural Versus Non-Epidural Pain Management After Minimally Invasive Esophagectomy: A Real-Life, High-Case-Load Center Experience

**DOI:** 10.3390/jcm13247669

**Published:** 2024-12-16

**Authors:** Sebastian Boehler, Markus Huber, Patrick Y. Wuethrich, Christian M. Beilstein, Stefano M. Arigoni, Marc A. Furrer, Yves Borbély, Dominique Engel

**Affiliations:** 1 Department of Anaesthesiology and Pain Medicine, Inselspital, Bern University Hospital, University of Bern, CH-3010 Bern, Switzerlandpatrick.wuethrich@insel.ch (P.Y.W.); christian.beilstein@insel.ch (C.M.B.);; 2 Department of Urology, Solothurner Spitäler AG, Kantonsspital Olten and Bürgerspital Solothurn, University of Bern, CH-4600 Olten, Switzerland; 3 Department of Visceral Surgery and Medicine, Bern University Hospital, University of Bern, CH-3010 Bern, Switzerland; yves.borbely@insel.ch

**Keywords:** minimally invasive esophagectomy, pain management, paravertebral block, thoracic epidural anesthesia, transversus abdominis plane catheter, patient-controlled analgesia

## Abstract

**Background/Objectives**: Esophagectomy is a key component of esophageal cancer treatment, with minimally invasive esophagectomy (MIE) increasingly replacing open esophagectomy (OE). Effective postoperative pain management can be achieved through various analgesic modalities. This study compares the efficacy of thoracic epidural anesthesia (TEA) with non-TEA methods in managing postoperative pain following MIE. **Methods**: A retrospective review was conducted on 110 patients who underwent MIE between 2018 and 2023. 1. TEA vs. 2. intravenous patient-controlled analgesia (PCA) alone vs. 3. transversus abdominis plane (TAP) catheter with PCA vs. 4. single-shot TAP block with paravertebral catheter (PVB) in combination with PCA were compared. The primary outcome was postoperative pain within the first 72 h, assessed using the numeric rating scale. Secondary outcomes included postoperative surgical complications (Clavien–Dindo classification (CDC)), patient satisfaction, and duration of induction and emergence, among others. **Results**: The incidence of an NRS > 3 during movement was 47.1%, 51%, 60.1%, and 48.3% for TEA, PCA alone, TAP + PCA, and PVB + PCA, respectively. For pain at rest, the rates were 8.3%, 4.3%, 11.2%, and 5%, respectively. High surgical complication rates were observed across all groups (CDC IIIa-V 31.6% overall), with patient satisfaction similarly high, regardless of the analgesic modality used (85% satisfied or very satisfied). No differences in the other secondary outcomes were observed. **Conclusions**: PVB combined with PCA offered analgesic efficacy and patient satisfaction comparable to TEA in managing postoperative pain following MIE.

## 1. Introduction

Esophageal cancer remains a commonly diagnosed, highly aggressive type of cancer, leading to more than 400.000 deaths per year globally [1]. For T2 and more advanced stages, surgical removal combined with neoadjuvant therapy is the standard of care [2]. Open esophagectomy (OE) is a high-risk thoraco-abdominal procedure, often associated with severe postoperative pain and a high incidence of complications [3]. Hence, thoracic epidural anesthesia (TEA) has been accepted as the gold standard for perioperative pain management [4], as postoperative pain leading to the inability to breathe without restriction has been identified as a contributor to the development of pulmonary complications. This, in turn, results in increased nursing costs and prolonged hospitalization [5]. However, a meta-analysis involving 891 patients challenged the use of TEA, as it did not provide superior outcomes over other analgesic regimens with regard to a reduction in pain or pulmonary complications [6]. Consequently, the 2015 PROSPECT guidelines for thoracotomy recommend prioritizing the use of a paravertebral catheter over TEA due to its lower rate of complications [7].

In recent years, advances in surgery led to a change to a minimally invasive form of esophagectomy (MIE). This transition was accompanied by improved postoperative outcomes with regard to hemorrhages and respiratory complications, as well as tendencies to decreased perioperative mortality and comparable results for oncologic resection [8,9,10]. Due to the smaller incisions resulting in lower postoperative pain, the use of TEA in MIE has been a subject of ongoing debate [11]. The video-assisted paravertebral block (PVB) offers an improved risk–benefit profile. The current literature demonstrate comparable analgesia for both minimally invasive esophagectomy (MIE) and thoracotomy, with advantages such as direct visual placement, reduced hypotension, and less urinary retention [6,12,13,14].

The results from a very recently published randomized controlled trial (PEPMEN) with almost 200 patients concluded that both techniques, the TEA and the PVB, are effective and are useful in clinical practice [15].

Nevertheless, the incidence of postoperative complications continues to be significant, highlighting both the high-risk nature of the surgery and the underlying morbidity within this patient population. This underscores the necessity for a comprehensive preoperative evaluation to identify individuals at an elevated risk for perioperative complications and mortality [16].

With the introduction of MIE at our institution in 2018, different analgesic regimens have been used sequentially, utilizing systemic and regional anesthesia as well as combinations thereof. In this study, we retrospectively analyzed to what extent different non-TEA techniques and TEA compare in postoperative analgesia for MIE as well as the practical considerations thereof.

## 2. Patients and Methods

This retrospective observational study reports on 110 patients from a single tertiary high-case-load center and is in accordance with the Strengthening the Reporting of Observational Studies in Epidemiology (STROBE) statement [17]. The Ethics Committee of the Canton of Bern, Switzerland (KEKBE 2021-00729, Chair Professor C. Seiler), granted ethical approval on 6 May 2021. The need for informed consent was waived.

All patient data were retrospectively evaluated and extracted from a maintained database that fully complies with the legal requirements of the Swiss federal act on research involving human beings. All patients who underwent elective MIE with dual-field lymphadenectomy, gastric conduit reconstruction, and cervical anastomosis (minimally invasive McKeown esophagectomy [18]) from August 2018 to March 2023 in our institution were primarily included. Four main approaches of perioperative analgesia were used in these patients: 1. TEA, 2. patient-controlled analgesia (PCA) only, 3. transversus abdominal plane catheter and PCA (TAP + PCA), and 4. single-shot bilateral TAP block, surgical paravertebral catheter, and PCA (PVB + PCA).

Patients who were unable to respond to the NRS questionnaire due to postoperative intubation and sedation, as well as those who did not receive any of the above specified analgesic regimens, were excluded.

### 2.1. Primary Outcome

The primary outcome was the numeric pain rating score (NRS), recorded at rest and with movement during the first 72 h after surgery. The inquiry of the NRS was conducted by our trained pain staff during visitation and on call. The patients were requested to rate their pain at rest and in movement on the numeric rating scale from zero to ten, based on their subjective sensation. During weekdays every patient is regularly visited at least once per day; during weekends and holidays this service was not always provided on a regular basis. For these reasons, NRS observations were taken at irregular intervals in the postoperative period for a maximum of 72 h.

### 2.2. Secondary Outcome

Secondary outcomes were postoperative in-hospital complications, according to the Clavien–Dindo classification (CDC) [19], patient satisfaction, number of visits per group, duration of induction and emergence, consumption of opioids and catecholamines intraoperatively, as well as fluid administration during surgery. Postoperative complications were graded using the CDC based on the required intervention. Grade I and II were treated pharmacologically, while Grade IIIa–V include complications with the necessity of interventions, life-threatening complications, and single-to-multiorgan dysfunction and death. Patient satisfaction with analgesic treatment was inquired by the pain staff on the final visitation. Patients were asked to rate their experience with the pain management as “not satisfied”, “satisfied”, and “very satisfied”. The treating anesthesiologist was in charge of the intraoperative hemodynamic management. A mean arterial pressure of 65 mmHg was aimed for, guided by invasive arterial pressure (and pulse pressure variation (goal PPV < 10)) and central venous pressure (6–12 mmHg) monitoring. Catecholamines or fluids were given accordingly. Further, we recorded days until the return of postoperative bowel function, removal of the bladder catheter, length of stay in the intermediate care unit (ICU), and length of hospital stay. In order to detect problems and side effects of the installed pain treatments, all written entries from the pain staff were analyzed. All irregularities were categorized into four groups: respiratory compromise, hypotension, nausea, and catheter dysfunction.

### 2.3. Operative Procedure

General anesthesia was induced intravenously, and then patients were intubated using a single-lumen endotracheal tube. For hemodynamic monitoring and drug administration, arterial and central venous cannulation was performed. After placement of a bronchial block in the right main bronchus, patients were placed in a left lateral-prone position (30° angle). Three trocars (tip of the scapula, ninth intercostal space subscapular line, and fifth intercostal space midaxillary line) with a capnothorax of 6 mmHg were used. The parietal pleura was incised, and the esophagus was prepared and encircled. The azygous vein was stapled, and the vagal nerves were transected bilaterally. Subcarinal and periesophageal lymphatic tissue, including the clipped thoracic duct, were dissected and maintained with the specimen up to the cervical level. Thoracic drains (20 F bilaterally) and paravertebral catheters were placed under visual guidance. Patients were then placed in a lithotomy position. Five trocars (10 mm epigastric median, right and left paramedian; 5 mm left subcostal and subxyphoidal) and a pneumoperitoneum of 12 mmHg were used. Preserving the gastroepiploic arcade, the gastrocolic ligament was opened and the stomach dissected away from the pancreas. The lesser gastric curvature and the left gastric artery were dissected and stapled off to create a tubular gastric conduit. The tip was sutured to the specimen. A neck incision of 4 cm at the anterior border of the sternocleidomastoid muscle was made, the esophagus mobilized, and the specimen together with the conduit pulled up. An esophago-gastrostomy was sutured over a nasogastric tube. To end the procedure, the hiatal opening was closed.

### 2.4. Catheter Insertion and Perioperative Pain Management

- For TEA (Group: TEA): The thoracic epidural catheter was placed in the interspace T5-7 prior to induction of anesthesia. The insertion site was determined using the classic landmark method, identifying the spinous process of T7 at the line crossing the inferior tip of the scapulae in the sitting position. An 18-gauge epidural needle was inserted through a paramedian or median approach, and the epidural space was identified using the loss of resistance technique. A test dose of 1.5 mL lidocaine 20 mg/mL with 0.005 mg/mL epinephrine was administered to rule out subarachnoid or intravascular placement. The TEA was then started with bupivacaine 2.5 mg/mL (bupivacaine 0.25% Bioren; Sintetica, Bioren, Switzerland) at a rate of 6-to-10 mL/h. No opioids were administered epidurally during the procedure. At the end of surgery, continuous epidural analgesia was maintained with an epidural mixture of bupivacaine 1 mg/mL, fentanyl 2 ug/mL, and epinephrine 2 ug/mL, using a CADD Legacy ambulatory infusion pump (model 6300; Deltec Inc., St Paul, MN, USA). The initial infusion rate was 6–8 mL/h, with additional bolus volumes of 5 mL (lockout time: 1 h).
- For the TAP catheter and PCA or single PCA (Group: TAP + PCA or PCA): In the period after TEA, but before the implementation of the current standard described below, patients received postoperatively either a tunneled, left-sided TAP catheter combined with an intravenous PCA using a CADD Legacy ambulatory infusion pump (model 6300; Deltec Inc., St Paul, MN, USA) or just the PCA. A 0.2% ropivacaine solution (Ropivacaine Fresenius Kabi 2 mg/mL, Fresenius Kabi, Kriens, Switzerland) was administered through the TAP catheter at a rate of 8-to-12 mL/h immediately after completion of surgery. With the intravenous PCA patients received 0.2 mg Hydromorphone (Hydromorphone Sintetica 20 mg/100 mL, Sintetica Switzerland) per dose. A lockout time of 7 min was programmed.
- For single-shot TAP and the PVB catheter and PCA (Group: PVB + PCA): The current pain management at our institution for McKeown MIE consists of an ultrasound-guided bilateral transversus abdominis plane block with 20 mL of Ropivacain 0.375% (Ropivacaine Fresenius Kabil 7.5 mg/mL, Fresenius Kabi Switzerland) for the laparoscopic portion of the MIE. This is performed immediately after induction of anesthesia. After completion of the thoracosopic part of the surgery, the surgeon places a paravertebral catheter (PVB) under direct thoracosopic vision. A 0.25% ropivacaine solution (Ropivacaine Fresenius Kabi 2.5 mg/mL, Fresenius Kabi Switzerland) is then administered through the catheter at a rate of 8-to-12 mL/h. Postoperatively, patients are supplied with an intravenous PCA pump at the same rate as described above.
- Standard intraoperative pain management: Further intraoperative pain management was at the discretion of the anesthesiologist in charge. In the postoperative period, all patients without contraindications received 1 g of metamizole (MINALGIN Inj Lös 1 g/2 mL i.v., Streuli Pharma Uznach, Switzerland) every 8 h. Rescue analgesics in the postoperative period were administered as needed, and the infusion rates of local anesthetics (TEA, TAP, and PVB) were adjusted within the above ranges to pain and sensory (distribution) levels by a member of the pain staff as needed. All catheters were usually left in place for 3 days. Before removal of the catheter, the dose of local anesthetic was reduced to ensure sufficient overlap with the systemic pain medication.
- Rescue analgesia protocols: There were no defined rescue analgesia protocols. Rescue analgesia was administered in the most suitable form determined by our pain staff: In the TEA group, in the first step, the infusion rate was increased (up to 15 mL/h); in the second step the composition of the TEA solution was changed (rotation to a “forte-mixture” of bupivacaine 2.5 mg/mL, fentanyl 2 ug/mL, and epinephrine 2 ug/mL) to achieve enhanced sensory blockade; and finally, if still judged insufficient, an additional PCA was used. In groups containing a PCA as part of the regimen an increase in the bolus volume was considered. Additionally, ketamine infusions were used to provide sufficient analgesia.

### 2.5. Statistical Methods

We performed a complete case analysis without imputation. In terms of summary measures, categorical variables were summarized with counts and percentages. Numerical variables were summarized with medians and interquartile ranges otherwise.

Group comparisons across treatment groups of baseline characteristics, surgical characteristics, as well as satisfaction were computed with the chi-square test or Fisher’s exact test for categorical variables and with the Kruskall–Wallis test for numerical variables.

The time evolution of the primary outcome (NRS at rest and at movement as a function of the visiting hour after the end of anesthesia) were graphically displayed by means of a Local Polynomial Regression Fitting (LOESS). We dichotomized the numeric rating scale with a threshold of 3 and compared the risk of an NRS > 3 for the four treatment regimens. Their pairwise contrasts were modeled with a generalized linear mixed model with a random offset for each patient. As these results are considered exploratory, no *p*-value adjustment for multiple comparisons was performed.

With respect to the secondary outcomes’ time to first flatus (TTF), time to first bowel movement, time to first spontaneous micturition, length of stay in the ICU, and length of hospital stay, we log-transformed the outcomes and the statistical significance of the treatment group was assessed by means of a log-rank test in a linear regression framework. We further adjusted the analyses of the secondary outcomes to age and sex.

All analyses were performed with R version 4.0.2 [20].

## 3. Results

### 3.1. Participants

**Figure 1:**
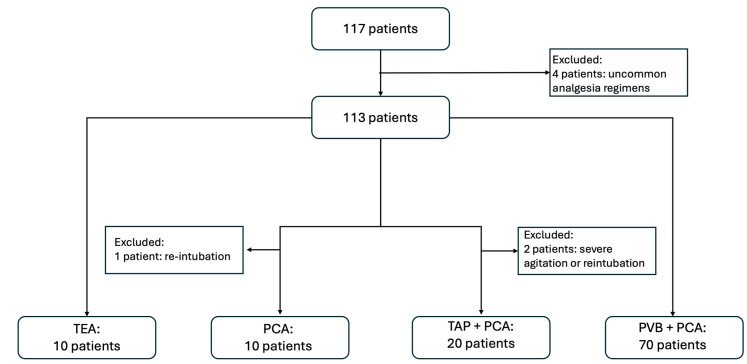
Flowchart of patient inclusion and exclusion.

Between August 2018 and March 2023, 117 patients underwent MIE at the University Hospital of Bern. Four patients receiving various combinations of analgesic regimens (e.g., TAP catheter + PVB catheter + PCA or PVB catheter + ketamine infusion) were excluded due to insufficient numbers (*n* = 3 and *n* = 1), which prevented comparability. Additionally, three patients were excluded because NRS assessment was not possible: one due to extreme agitation and two due to re-intubation and sedation for more than 72 h postoperatively (Figure 1). Finally, 110 patients were included for the final analysis.

### 3.2. Descriptive and Outcome Data

Baseline characteristics of the 110 included patients are shown in Table 1. On average, our pain staff conducted four visitations per patient within 72 h postoperatively.

### 3.3. Primary Outcomes

**Figure 2:**
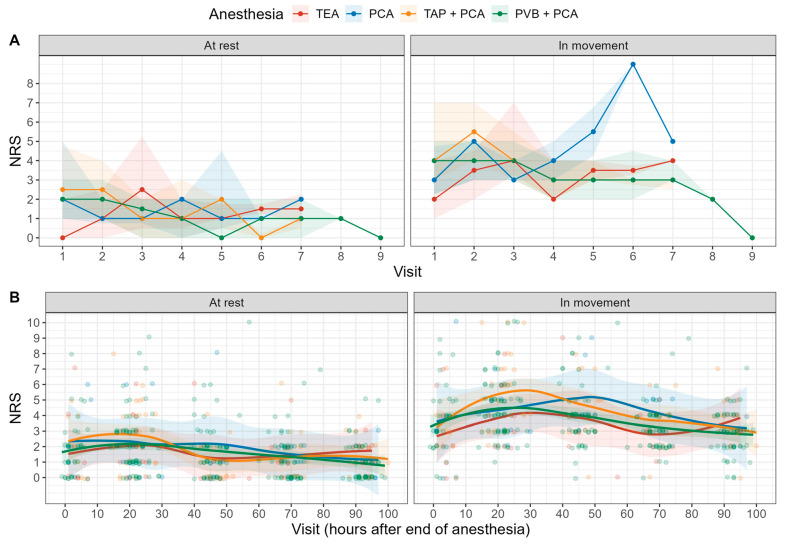
NRSs in movement and at rest; (**A**) shows the queried NRSs for each visit; (**B**) shows NRSs over time.

Our study did not find any significant difference in pain scores at rest or during movement between the four groups within the first 72 h after surgery (Figure 2). The NRSs in movement during the third visitation (usually on postoperative day (POD) 2) were 4 [3;7], 3 [3;4], 4 [3;6], and 4 [3;5] for TEA, PCA, TAP + PCA, and PVB + PCA, respectively. The incidence of an NRS > 3 during movement was 47.1%, 51%, 60.1%, and 48.3% for TEA, PCA, TAP + PCA, and PVB + PCA, respectively. For pain at rest, rates were 8.3%, 4.3%, 11.2%, and 5%, respectively. No significant difference was observed in the risk of high pain (NRS > 3) at rest or during movement (Table 2). Both TEA and PVB + PCA appear to result in lower median NRSs during movement within the first 48 h when compared to the other strategies.

### 3.4. Secondary Outcomes

Throughout all groups the surgical complication rate during hospitalization was high (with minor (CDC I-II) and major (CDC IIIa-V) complications occurring in 25.8% and 31.6% of patients, respectively, with no significant difference between the groups (Table 3)). Patient satisfaction was generally good (85% satisfied or very satisfied). We observed no difference in the duration of induction (mean 32 min, 32–38 min), while there was a trend to a shorter emergence time in the PCA group (mean 34 min vs. 24 min in PCA). Intraoperatively there was a trend to lower opioid consumption (mean 600 mcg vs. 425 mcg Fentanyl in TEA) and to higher vasopressor consumption (mean 18 mcg/kg vs. 30 mcg/kg Norepinephrine) in the TEA group. Intraoperative fluid administration, time to first spontaneous miction and bowel movements, length of the ICU (mean 2 days), as well as length of the hospital stay (mean 15 days) did not differ between groups. The full data of secondary outcomes can be seen in Table 3, Table 4 and Table 5.

Respiratory compromise included all types of subjective and objective breathing difficulties, including disability of coughing and inability of deep inspiration. Catheter-associated problems that led to an interruption of the medication for more than an hour or to catheter removal were included. Side effects of the different pain treatments are shown in Table 6. The PVB + PCA group was associated with less episodes of hypotension and less impaired breathing.

One patient in the TEA group received a PCA and subsequently a TEA was inserted due to catheter dislocation. Five patients in the PVB group received an additional ketamine infusion as rescue analgesia. There were no conclusive data on how many patients with a PCA received an augmentation of the intravenous bolus.

## 4. Discussion

The findings of our study indicate no statistically significant differences in pain scores at rest or during movement across the four groups (TEA, PCA, TAP + PCA, and PVB + PCA) within the first 72 h postoperatively. Similarly, the risk of experiencing high pain (NRS > 3) did not differ significantly between groups. Although not statistically significant, both TEA and PVB + PCA were associated with lower median NRSs during movement in the first 48 h, suggesting a potential early advantage over the other analgesic strategies. This is comparable with findings of the recently published PEPMEN trial [15].

Following the transition from OE to MIE, the most adequate method in terms of the risk–benefit ratio for perioperative analgesia was investigated. Various reasons motivated the change from the TEA, including high rates of failure, potential serious epidural complications, urinary retention, and perioperative hypotension [11,21]. In the early phase after the TEA, initially a PCA-only strategy was used, and later a combination of postoperatively inserted TAP catheters in combination with PCA for postoperative analgesia was used. This resulted in higher postoperative pain scores in the PCA-only group and a noticeable increase in catheter-associated complications in the TAP catheter group, particularly leakage from the catheter site, leading to discontinuation of its use. With the current regimen of PVB + PCA, we observed postoperative pain levels comparable to TEA, but with fewer catheter-associated complications than in the TAP catheter group. Previous studies support the analgesic efficacy of surgically inserted PVB [13,14]. The four regimens were used sequentially and adapted over varying time intervals according to the experience made by surgeons and anesthesiologists. No patients were a priori assigned to any group, but rather treated according to the current standard operating procedure, which changed over time, explaining the different group sizes.

We found that patients with TEA had the lowest NRSs in the direct postoperative phase, despite receiving less intraoperative opioids. However, this effect was neutralized by the second evaluation on the first postoperative day. These results are similar to those described by Feenstra in 2021 [14]. This increase in pain could be associated with the routine transfer from the ICU to the surgical ward on the first postoperative day, which requires the patients to be hemodynamically stable. The before-described hypotension caused by TEA is often managed by a reduction in the epidural infusion rate, albeit leading to a higher NRS.

Another reason why TEA could not show significantly lower NRSs in our study was the performed McKeown procedure. The incision used for the cervical gastroesophageal anastomosis is made above the dermatomes covered by a TEA or PVB [18]. Therefore, only systemic analgesia including an additional PCA could provide pain relief in this area.

Half of the patients in the TEA group developed respiratory distress; this is more than any other group. This is comparable with the results of Kingma et. al, where they described an effective pain control at the price of a high sensory blockade of TEA in esophagectomy in about 50% of cases [11]. However, there might be a bias, due to the subjective recording of our pain staff: neurological examination and survey of impaired breathing is more strictly performed and recorded in patients with TEA compared to patients with non-TEA pain regimens.

In our study, we further found a high risk of postoperative surgical complications measured by the CDC across all groups, with no significant differences noted (Table 4), and 32% of our patients developed a major complication (Clavien–Dindo 3a to 5); however, the role of the analgesic regimen seems to be negligible.

Patient satisfaction was generally high, with 85% reporting satisfaction. Very similar results are seen in the PEPMEN trial [15]; however, three patients in the PVB group were dissatisfied due to insufficient analgesia or catheter site issues, while one in the TEA group experienced a “patchy” epidural. Our pain team highlighted respiratory compromise and catheter dysfunction as the most frequent problems in the postoperative period, with hypotension potentially underreported as it was not the primary focus of the staff. Although a tunneled approach was used, most catheter-associated problems (leakage, disconnection, dislocation, and inadvertent dislodgement) were found in the TAP + PCA group, most probably due to the lateral abdominal site, which finally led to the abandonment of the technique.

This study has several limitations. Primarily, these are the uneven distribution of patients among the groups, lack of blinding, and variability in the frequency and timing of visitations, limiting the proper interpretation of the data presented. Additionally, due to limitations in our clinical information system, the placement of TEA was not included in the induction time, although all other catheters placed under general anesthesia were accounted for. Based on our clinical experience, TEA placement typically requires an additional 20 min, which should be factored into the reported induction time. As our primary outcome was based on NRSs, we excluded patients unable to report their pain. Lastly, the retrospective design introduces the possibility of unrecorded complications.

Potential confounding factors include pre-existing pain and mental disorders, the duration and complexity of the operative procedure, as well as the timing of the visitation and NRS inquiry. However, all the mentioned factors depict the clinical difficulties of perioperative pain management under real-life circumstances.

One strength of this study is seen within the consistency on the part of surgeons. All 110 patients were operated on by the same surgeon. Another strength lies in the direct comparison of different pain regimens, including two non-TEA catheters (TAP and PVB), which were conducted by trained anesthesiologists and carried on by our pain staff. To the best of our knowledge this is the first study that compared TEA, PVB, TAP, and PCA.

This retrospective study was unable to identify the most appropriate postoperative analgesic techniques, partly due to the unequal distribution of patients across the groups. Nevertheless, PVB combined with a systemic PCA constitutes a safe and effective form of postoperative analgesia, with a well-balanced risk–benefit profile. Further, the recent introduction of multimodal anesthesia with magnesium, dexmedetomidine, and ketamine will probably further increase the efficacy of the current regimen. Finally, randomized controlled trials are needed to outline the findings.

## Figures and Tables

**Table 1:** Patient characteristics.

	ALL	TEA	PCA	TAP + PCA	PVB + PCA	*p*
	*n* = 110	*n* = 10	*n* = 10	*n* = 20	*n* = 70	
**Sex** (female)	22 (20%)	4 (40%)	3 (30%)	4 (20%)	11 (16%)	0.258
**Age** (yrs, [IQR])	64 [58;69]	67 [63;71]	66 [64;71]	63 [56;72]	64 [57;68]	0.215
**BMI** (kg · m^−2^ [IQR])	26 [22;28]	24 [21;28]	25 [24;28]	25 [22;29]	26 [22;28]	0.902
**ASA status**						0.630
2	7 (6%)	1 (10%)	1 (10%)	0 (0%)	5 (7%)	
3	94 (85%)	7 (70%)	8 (80%)	19 (95%)	60 (86%)	
4	9 (8%)	2 (20%)	1 (10%)	1 (5%)	5 (7%)	
**CHD** (Yes)	16 (15%)	0 (0%)	4 (40%)	5 (25%)	7 (10%)	0.021
**Hypertension** (Yes)	54 (49%)	5 (50%)	7 (70%)	11 (55%)	31 (44%)	0.476
**COPD**						0.338
GOLD 2	1 (1%)	1 (10%)	0 (0%)	0 (0%)	0 (0%)	
GOLD 3	1 (1%)	0 (0%)	0 (0%)	0 (0%)	1 (1%)	
None	108 (98%)	9 (90%)	10 (100%)	20 (100%)	69 (99%)	
**OSAS** (Yes)	8 (7%)	1 (10%)	1 (10%)	2 (10%)	4 (6%)	0.941
**Metabolism**						0.090
None	90 (82%)	10 (100%)	6 (60%)	18 (90%)	56 (80%)	
IDDM/NIDDM	20 (18%)	0 (0%)	4 (40%)	2 (10%)	14 (20%)	
CKD *	16 (15%)	1 (10%)	2 (20%)	3 (15%)	10 (14%)	>0.99

Abbreviations: BMI—body mass index; ASA—American Society of Anaesthesiologists; CHD—coronary heart disease; COPD—chronic obstructive pulmonary disease; GOLD—Global Initiative For Chronic Obstructive Lung Disease; OSAS—obstructive sleep apnoea syndrome; IDDM/IDDM non/insulin-dependent diabetes mellitus. * Chronic kidney disease, defined as GFR < 60 mL/kg/min.

**Table 2:** Risk of an NRS > 3 in movement and at rest and between-group differences in risk of an NRS > 3 in movement and at rest.

Regimen	Risk of NRS > 3 (in Movement)	Risk of NRS > 3 (at Rest)
TEA	47.1% (95%-CI: 22.9–71.2%)	8.3% (95%-CI: −3.2–19.8%)
PCA	51.0% (95%-CI: 24.1–77.8%)	4.3% (95%-CI: −4.6–13.1%)
TAP + PCA	60.1% (95%-CI: 43.4–76.7%)	11.2% (95%-CI: 0.5–21.8%)
PVB + PCA	48.3% (95%-CI: 39.4–57.3%)	5.0% (95%-CI: 1.1–9.0%)
**Contrasts**	Differences in risk of NRS > 3 (in movement)	Differences in risk of NRS > 3 (at rest)
TEA vs. PCA	−3.9% (95%-CI: −51.1–43.3%, *p* > 0.99)	4.1% (95%-CI: −14.2–22.4%, *p* = 0.941)
TEA vs. TAP + PCA	−13.0% (95%-CI: −51.5–25.6%, *p* = 0.823)	−2.8% (95%-CI: −22.5–16.9%, *p* = 0.983)
TEA vs. PVB + PCA	−1.3% (95%-CI: −35.0–32.4%, *p* > 0.99)	3.3% (95%-CI: −11.7–18.3%, *p* = 0.943)
PCA vs. TAP + PCA	−9.1% (95%-CI: −50.5–32.3%, *p* = 0.943)	−6.9% (95%-CI: −24.2–10.4%, *p* = 0.734)
PCA vs. PVB + PCA	2.6% (95%-CI: −34.4–39.7%, *p* > 0.99)	−0.8% (95%-CI: −12.4–10.8%, *p* > 0.99)
TAP + PCA vs. PVB + PCA	11.7% (95%-CI: −13.1–36.5%, *p* = 0.619)	6.1% (95%-CI: −7.6–19.9%, *p* = 0.661)

**Table 3:** Perioperative data: induction and emergence time, intraoperative opioid, fluid, blood products, catecholamine administration, and complications.

	ALL	TEA	PCA	TAP + PCA	PVB + PCA	*p*
	*n* = 110	*n* = 10	*n* = 10	*n* = 20	*n* = 70	
**Induction** (min [IQR]))	32 [26;46]	37 [24;47]	40 [32;60]	32 [26;42]	32 [26;44]	0.458
**Emergence** (min [IQR]))	35 [24;53]	31 [20;37]	26 [20;45]	52 [32;60]	34 [24;54]	0.037
**Fentanyl** (mcg/kg [IQR])	8 [6;11]	6 [5;8]	9 [6;11]	9 [7;11]	8 [7;11]	0.079
**Hydromorphon** (Yes)	16 (15%)	1 (10%)	2 (20%)	3 (15%)	10 (14%)	>0.99
**Methadon** (Yes)	6 (5%)	0 (0%)	1 (10%)	0 (0%)	5 (7%)	0.447
**Remifentanil** (Yes)	15 (14%)	1 (10%)	2 (20%)	3 (15%)	9 (13%)	0.962
**Ringer’s lactate** (mL)	2125 [1900;3000]	2225 [1700;2925]	2150 [2000;2875]	1950 [1300;3000]	2200 [1925;3000]	0.357
**RCC** (Yes):	1 (1%)	0 (0%)	0 (0%)	0 (0%)	1 (1%)	>0.99
**FFP** (Yes):	9 (8%)	2 (20%)	1 (10%)	0 (0%)	6 (9%)	0.282
**TC** (No)	110 (100%)	10 (100%)	10 (100%)	20 (100%)	70 (100%)	
**Norepinephrine** (mcg/kg [IQR])	18 [13;26]	30 [16;46]	16 [13;18]	15 [9;24]	19 [14;26]	0.086
**Clavien–Dindo**						0.669
0	40 (36%)	5 (50%)	2 (20%)	6 (30%)	27 (39%)	
1	6 (5%)	0 (0%)	1 (10%)	3 (15%)	2 (3%)	
2	23 (21%)	1 (10%)	1 (10%)	7 (35%)	14 (20%)	
3a	13 (12%)	0 (0%)	3 (30%)	1 (5%)	9 (13%)	
3b	9 (8%)	1 (10%)	1 (10%)	1 (5%)	6 (9%)	
4	1 (1%)	0 (0%)	0 (0%)	0 (0%)	1 (1%)	
4a	1 (1%)	0 (0%)	0 (0%)	0 (0%)	1 (1%)	
4b	8 (7%)	1 (10%)	1 (10%)	0 (0%)	6 (9%)	
5	3 (3%)	1 (10%)	0 (0%)	0 (0%)	2 (3%)	

RCC = red cell concentrate; FFP = fresh frozen plasma; TC = thrombocyte concentrate; IQR = interquartile range.

**Table 4:** Satisfaction with pain management and numbers of NRS queries.

	All	TEA	PCA	TAP + PCA	PVB + PCA	*p*
	*n* = 110	*n* = 10	*n* = 10	*n* = 20	*n* = 70	
**Satisfaction**:						0.806
Not assessed	11 (10%)	1 (10%)	2 (20%)	3 (15%)	5 (7%)	
Not satisfied	4 (4%)	1 (10%)	0 (0%)	0 (0%)	3 (4%)	
Satisfied	25 (23%)	1 (10%)	2 (20%)	4 (20%)	18 (26%)	
Very satisfied	70 (64%)	7 (70%)	6 (60%)	13 (65%)	44 (63%)	
**Number of visits**						
At rest	4 [3;5]	4 [2;5]	2 [2;4]	4 [3;5]	4 [3;5]	0.222
In movement	4 [3;5]	4 [2;5]	2 [2;3]	4 [3;5]	4 [3;5]	0.067

**Table 5:** Time to first flatus, time to first bowel movement, time to first spontaneous micturition (successful trial of void), length of stay in ICU, and length of hospital stay.

	ALL	TEA	PCA	TAP + PCA	PVB + PCA	*p* ^†^	N
	*n* = 110	*n* = 10	*n* = 10	*n* = 20	*n* = 70		
**Time to first flatus** (days)	5 [4;6]	6 [4;6]	4 [3;6]	4 [4;5]	5 [4;7]	0.994	73
**Time to first BM** (days)	5 [4;6]	6 [3;6]	5 [4;6]	5 [4;6]	6 [4;7]	0.606	94
**Time to first micturition** (days)	5 [3;7]	6 [2;6]	6 [4;8]	6 [4;7]	5 [3;7]	0.478	81
**LOS ICU** (days)	2 [1;4]	2 [1;3]	3 [1;5]	2 [1;3]	2 [1;4]	0.688	94
**LOS Hospital** (days)	15 [12;20]	15 [9;18]	14 [13;26]	14 [11;15]	15 [12;20]	0.518	95

† Adjusted linear regression models with a log-transformed outcome based on a likelihood ratio test of age and sex; BM = bowel movement, LOS = length of stay, ICU = immediate care unit.

**Table 6:** Undesired effects of the analgesic modality.

	All	TEA	PCA	TAP + PCA	PVB + PCA	*p*
	*n* = 110	*n* = 10	*n* = 10	*n* = 20	*n* = 70	
**Undesired effects**						0.256
Not present	68 (61.8%)	5 (50.0%)	7 (70.0%)	9 (45.0%)	47 (67.1%)	
Present	42 (38.2%)	5 (50.0%)	3 (30.0%)	11 (55.0%)	23 (32.9%)	
**Type** (multiple possible):	N = 54	N = 10	N = 3	N = 14	N = 27	0.051
impaired breathing	28 (51.9%)	5 (50.0%)	2 (66.7%)	5 (35.7%)	16 (59.3%)	
hypotension	4 (7.4%)	3 (30.0%)	0 (0.0%)	1 (7.1%)	0 (0.0%)	
nausea	3 (5.6%)	0 (0.0%)	1 (33.3%)	1 (7.1%)	1 (3.7%)	
catheter or pump dysfunction	19 (35.2%)	2 (20.0%)	0 (0.0%)	7 (50.0%)	10 (37.0%)	

## Data Availability

Data will be provided upon request.
